# Soft skills as core competencies: a decade of relationship-based pediatric antimicrobial stewardship in Colombia

**DOI:** 10.1017/ash.2026.10903

**Published:** 2026-10-08

**Authors:** Juan Pablo Londono-Ruiz, Ana María Monsalve-Quintero, Iván Felipe Gutierrez-Tobar

**Affiliations:** 1 Clínica Reina Sofia Pediátrica y Mujer, Clínicas Colsanitas, Grupo Keralty, Colombia; 2 Clínica Infantil Colsubsidio, Colombia; 3 Clínica Imbanaco, Colombia; 4 Universidad del Valle, Cali, Colombia; 5 Clínica Infantil Santa Maria del Lago, Clínicas Colsanitas, Grupo Keralty, Bogota, Colombia

## Abstract

Antimicrobial stewardship programs are usually judged by technical outputs, yet they most often succeed or fail at the human interface. We describe nearly a decade of experience at a pediatric tertiary hospital in Bogotá, Colombia, where a relationship-based program—real-time prospective audit with daily, in-person feedback rather than hard restriction—was paired with deliberate education (weekly “PROA tips,” syndrome-specific algorithms, and explicit macrolide-use criteria). Against a 2016 baseline, hospitalwide annual days of therapy (DOT) for ceftriaxone, cefepime, meropenem, and vancomycin fell by approximately 26%–73%. These reductions were maintained through 2020, when COVID-19 disrupted in-person rounds: ceftriaxone and cefepime continued to decline, and although meropenem and vancomycin rose modestly, neither returned to preprogram levels, with the downward trend resuming in 2021. The education-led macrolide intervention reduced clarithromycin DOT by approximately 73% between 2019 and 2020 and by more than 90% by 2022, while guideline-concordant prescribing rose from 45% to over 90%. Our experience suggests that behavioral competencies—communication, persuasion, negotiation, and relational leadership—function as the core of stewardship rather than as adjuncts. Enablement and in-person feedback, not restriction, sustained behavior change and protected program gains through a major disruption; education was effective when embedded in practice and linked to feedback. These observations are from a single center and are uncontrolled, but they suggest that investing in the human skills of stewardship—and teaching, recruiting for, and measuring them—may help similar programs, particularly in pediatric and resource-limited settings.

## Context

Antimicrobial stewardship programs (ASPs) are traditionally valuated by quantitative and technical endpoints, yet their clinical success hinges on the human interface—the bedside conversation, the nuanced negotiation with a treating prescriber, and the delicate moment a recommendation is accepted, adapted, or quietly ignored. The *Guidance for the Knowledge and Skills Required for Antimicrobial Stewardship Leaders* (endorsed by the Society for Healthcare Epidemiology of America [SHEA], the Infectious Diseases Society of America [IDSA], the Pediatric Infectious Diseases Society [PIDS], and the Society of Infectious Diseases Pharmacists [SIDP]) formally incorporates change management, effective interpersonal communication, conflict resolution, and data visualization among the essential core competencies required to direct an ASP.^1^ These “soft skills” are especially decisive in pediatrics and resource-limited settings, where formal infectious diseases specialist staffing is scarce and programs must rely on interprofessional collaboration and professional trust rather than top-down administrative authority.

## Problem

At a tertiary children’s hospital in Bogotá, Colombia, we sought to curb the overuse of broad-spectrum antimicrobials (primarily third- and fourth-generation cephalosporins, carbapenems, and glycopeptides) and macrolides. While formulary restriction and prior-authorization policies represent standard, effective stewardship strategies globally, they can inadvertently generate clinical friction, administrative fatigue, and transactional barriers in resource-constrained environments.^2^ The challenge was whether a relationship-based program built on communication, feedback, and education could lower and sustain antimicrobial consumption in a setting with limited specialist staffing.

## How we addressed it

Rather than relying on restrictive prior-authorization policies which, while effective in many settings, can risk friction in early program stages, we implemented real-time prospective audit with daily, in-person feedback delivered by the stewardship team—an approach modeled on relationship-based “handshake” stewardship.^3^ Recommendations were framed as clinical decision support and safety optimization rather than hierarchical policing: stewards conducted in-person ward rounds alongside treating pediatricians, engaging in collaborative, case-based discussions to reach consensus before documenting agreed-upon modifications in the electronic medical record. To navigate the local hierarchy and prescribing etiquette—where unwritten clinical autonomy and senior consultant preferences heavily influence decisions—stewards adopted an enablement strategy. We operationalized this through: department-specific and hospitalwide antimicrobial consumption dashboards were shared directly with clinical teams and hospital leadership, and we distributed weekly institutional micro-learning bulletins (designated locally as “PROA tips,” from the Spanish acronym for ASPs) addressing practical pearls, dosing optimization, and diagnostic stewardship; also, syndrome-specific algorithms and infographics, and explicit institutional criteria for macrolide use. This approach respected prescriber autonomy while systematically aligning bedside choices with institutional evidence.^4^

## Scientific learnings

**Figure 1.**
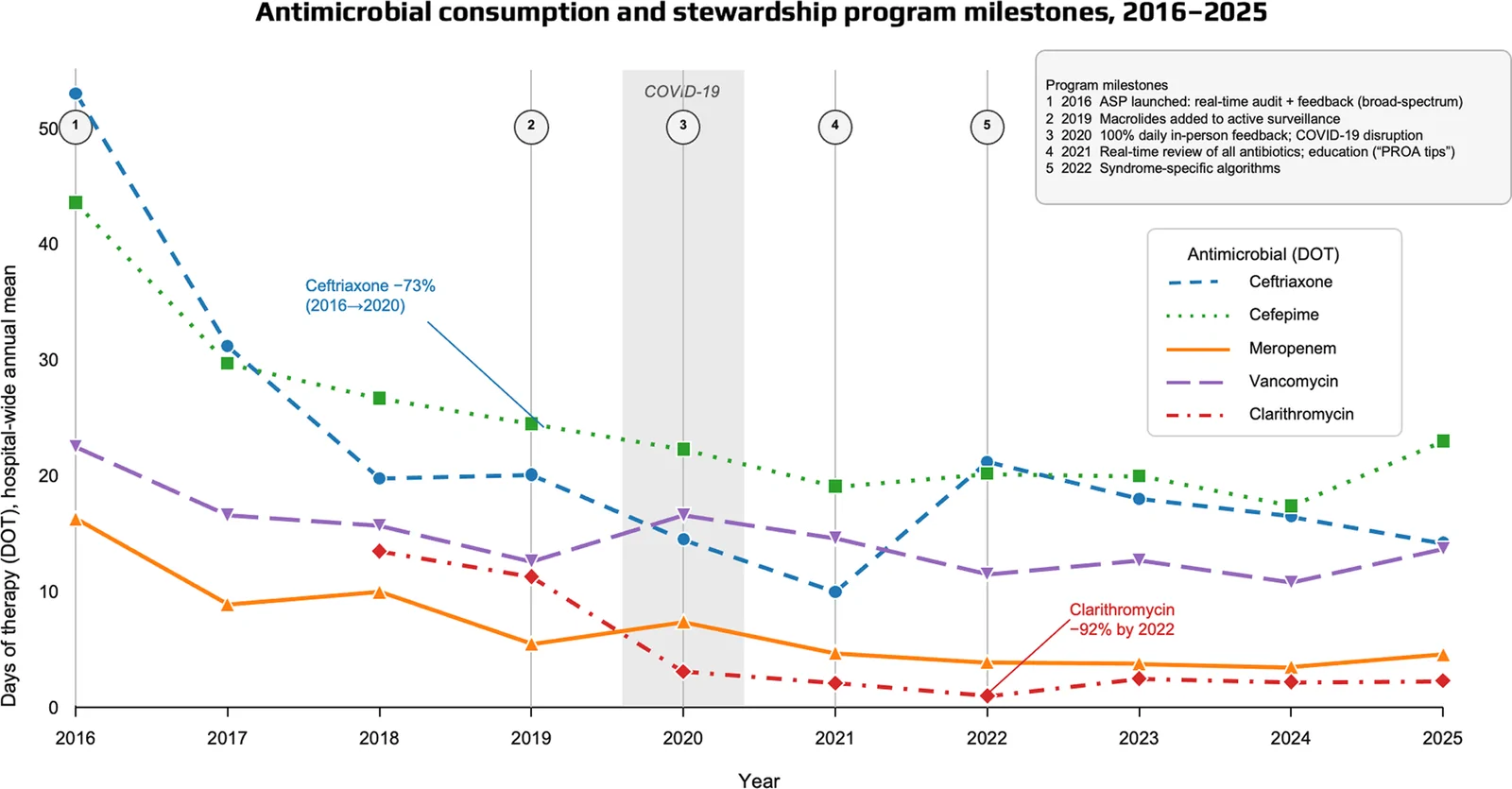
Figure 1 long description. Hospital-wide annual antimicrobial days of therapy (DOT) for four broad-spectrum agents and clarithromycin, 2016–2025, with stewardship program milestones; the shaded band marks the 2020 COVID-19 disruption of in-person rounds.

Across nearly a decade of prospective surveillance (2016–2025), annual hospitalwide days of therapy (DOT) demonstrated sustained decreases across all targeted broad-spectrum agents compared to the 2016 baseline (Figure 1): ceftriaxone decreased by 73% (from 53.0 to 14.2 DOT), meropenem by 72% (from 16.3 to 4.6 DOT), cefepime by 48% (from 43.8 to 22.8 DOT), and vancomycin by 40% (from 22.6 to 13.6 DOT). The resilience of this relational model was tested during the 2020 COVID-19 pandemic, when in-person multidisciplinary rounds were temporarily disrupted and clinical teams faced severe staffing reallocations. Despite these constraints, ceftriaxone and cefepime utilization maintained a downward trajectory. While meropenem and vancomycin consumption experienced modest transient elevations under pandemic-related diagnostic uncertainty and critical care pressures, neither rebounded toward baseline preintervention levels, and prepandemic downward trends promptly resumed by 2021. The targeted macrolide initiative illustrated the synergistic mechanism of coupling education with real-time audit: following the implementation of institutional criteria and bedside feedback, clarithromycin DOT decreased by 78% between 2019 and 2020 and achieved an overall reduction exceeding 90% by 2022, accompanied by an increase in guideline-concordant prescribing from 45% to over 90%. Passive guideline distribution had achieved only modest, variable adherence initially; consistent with broader behavioral stewardship literature, the inflection point in prescribing patterns occurred only when work-based education was actively reinforced by daily prospective audit and feedback.^5^

## Leadership and management lessons

Our experience demonstrates that behavioral competencies—such as assertive communication, active listening, principled negotiation, and relational leadership—are not peripheral soft skills, but rather the core operating system of sustainable stewardship, aligning with recent international leadership frameworks.^6^ Three lessons stand out. First, prospective audit paired with respectful, in-person feedback established safety and professional trust. This relational foundation prevented behavioral regression and protected long-term program gains even during major organizational disruptions. Second, education works when it is embedded in practice and tied to feedback, not delivered as a one-off lecture. Third, these skills can be taught, recruited for, and measure. While DOT serve as the primary metric of programmatic impact, the underlying relational effectiveness of the team is directly reflected in process measures, notably high prescriber recommendation acceptance rates and sustained guideline concordance over time. For pediatric and resource-limited programs worldwide, the transferable message is simple: investing in the human skills of stewardship is what makes the technical work succeed.

## Transferable message

For pediatric and resource-limited stewardship programs worldwide, technical expertise alone is insufficient to sustain behavioral change. Investing in core relational competencies—assertive communication, principled negotiation, and daily enablement—is the definitive operational foundation that enables clinical and epidemiological interventions to succeed.

## Figure 1. Long description

Panel: A line graph depicting antimicrobial consumption and stewardship program milestones from 2016 to 2025. The horizontal axis represents the years from 2016 to 2025, and the vertical axis represents the days of therapy (DOT), hospital-wide annual mean. The graph includes five data lines representing different antimicrobials: Ceftriaxone, Cefepime, Meropenem, Vancomycin, and Clarithromycin. Each line shows the trend of antimicrobial usage over the years. Key milestones are marked with numbers and include the launch of the ASP in 2016, the addition of real-time audit and feedback in 2019, COVID-19 disruption in 2020, real-time review of all antibiotics in 2021, and the introduction of syndrome-specific algorithms in 2022. The shaded band indicates the COVID-19 disruption period in 2020.

Navigate back to Figure 1..
